# Gene Module Analysis Reveals Cell-Type Specificity and Potential Target Genes in Autism’s Pathogenesis

**DOI:** 10.3390/biomedicines9040410

**Published:** 2021-04-10

**Authors:** Guoli Ji, Shuchao Li, Lishan Ye, Jinting Guan

**Affiliations:** 1 Department of Automation, Xiamen University, Xiamen 361102, China; glji@xmu.edu.cn (G.J.); sclee@stu.xmu.edu.cn (S.L.); 2 National Institute for Data Science in Health and Medicine, Xiamen University, Xiamen 361102, China; 3 Xiamen Health and Medical Big Data Center, Xiamen 361008, China

**Keywords:** autism spectrum disorder, cell type-specific, gene module, matrix decomposition

## Abstract

Multiple genetic factors contribute to the pathogenesis of autism spectrum disorder (ASD), a kind of neurodevelopmental disorder. Genes were usually studied separately for their associations with ASD. However, genes associated with ASD do not act alone but interact with each other in a network module. The identification of these modules is the basis for the systematic understanding of the pathogenesis of ASD. Moreover, ASD is characterized by highly pathogenic heterogeneity, and gene modules associated with ASD are cell-type-specific. In this study, based on the single-nucleus RNA sequencing data of 41 post-mortem tissue samples from the prefrontal cortex and anterior cingulate cortex of 19 ASD patients and 16 control individuals, we applied sparse module activity factorization, a matrix decomposition method consistent with the multi-factor and heterogeneous characteristics of ASD pathogenesis, to identify cell-type-specific gene modules. Then, statistical procedures were performed to detect highly reproducible cell-type-specific ASD-associated gene modules. Through the enrichment analysis of cell markers, 31 cell-type-specific gene modules related to ASD were further screened out. These 31 gene modules are all enriched with curated ASD risk genes. Finally, we utilized the expression patterns of these cell-type-specific ASD-associated gene modules to build predictive models for ASD. The excellent predictive performance also proved the associations between these gene modules and ASD. Our study confirmed the multifactorial and cell-type-specific characteristics of ASD pathogeneses. The results showed that excitatory neurons such as L2/3, L4, and L5/6-CC play essential roles in ASD’s pathogenic processes. We identified the potential ASD target genes that act together in cell-type-specific modules, such as *NRG3*, *KCNIP4*, *BAI3*, *PTPRD*, *LRRTM4*, and *LINGO2* in the L2/3 gene modules. Our study offers new potential genomic targets for ASD and provides a novel method to study gene modules involved in the pathogenesis of ASD.

## 1. Introduction

Autism spectrum disorder (ASD) is a neurodevelopmental disorder, the core symptoms of which are difficulties in social interaction and communication, narrow interests, and repetitive behaviors. Current etiological studies believe that ASD is a complex mental illness with high heritability and etiological heterogeneity [1]. ASD was reported as a multi-system disorder involving genetics, immunogenetics, immunology, microbiology, metabolic, and so on [2]. Recent studies have shown that ASD originates from developmental disorders of the whole brain before and early postpartum, involving cell proliferation, neurogenesis, migration, growth of laminar tissue and neurites, spinal development in the late pregnancy and early postnatal period, and synapse occurrence and synaptic function [3]. With the deepening of genomic research, risk genes related to ASD have been found one after another. Recently, the number of such genes reached nearly a thousand. One of the most representative research findings is the Simons Foundation Autism Research Initiative (SFARI) database [4]. However, none of the genes has an absolute advantage in the treatment and clinical manifestation of ASD [4]. A recent review illustrated that the genome is essentially a dynamic interactive molecular network formed by DNA sequence that is sensitive to the environment, and the epigenome tunes the matching between information from outside and responding mechanisms programmed in genes [2]. This means that the combined action of multiple genes should be considered when studying the pathogenesis of ASD. Although ASD-related genes had traditionally been classified by their impacts on synapses or developmental processes [5,6], recent research has highlighted the interconnections between these two classifications. Synaptic genes, such as neurotransmitter receptors or synaptic scaffolds, are critical in developmental checkpoints, while chromatin modifiers, traditionally labeled as developmental, are essential for life-long synaptic homeostasis [7]. The latest literature survey of hundreds of published reports [3] found that ASD involves multiple prenatal development processes. Specifically, among 58 ASD-risk genes with typical neural functions, 33 (57%) were involved in proliferation, 15 (26%) were involved in migration and cell fate decisions, 30 (52%) were involved in neurite growth, and 34 (59%) were involved in synaptogenesis and synaptic function. About two-thirds of these genes influence two or more of the mentioned processes. Another new study found 102 hypothesized ASD-risk genes, 98 of which were most expressed in the prenatal cortex, including cortical, cerebellar, amygdala, hippocampus, and striatum regions, involving the formation of the prenatal cortex, while they are not strongly expressed after delivery [8]. In summary, how to accurately locate the disease-causing genes in the process of ASD and to explain the mechanism of ASD pathogenesis are still a massive challenge.

Although clinical and genetic heterogeneity in ASD has long been demonstrated, direct assessment of specific cell types in the brains of ASD individuals has only recently become feasible [9]. Studies of single-nucleus RNA sequencing in cortical tissues of patients with ASD have found that malfunctioning genes expressed in specific cell types of the neocortex are ideal candidates for the development of ASD-targeted therapies [10]. In a recent study [11], researchers attempted to identify the cell-type-specific changes of genes shared across cortical areas of ASD patients. They found that in three groups of ASD patients, the same 30 genes were upregulated in four cortical regions. These genes are expressed in adult microglia, astrocytes, and brain endothelial cells. The results showed that activation of astrocytes, microglia, and endothelial cells in the neocortex is a common feature of ASD pathology across multiple cortical regions, and astrocytes and microglia have been shown to regulate the formation and pruning of synapses during development [11]. Although the studies [9,11] have pointed out the cell specificity in the pathogenic factors of ASD, only the relationship between a single gene and ASD has been analyzed in the previous research process.

Considering gene interactions and cell-type specificity in ASD pathogenic factors, in this study, we proposed an analytical approach based on cell-type-specific gene modules to analyze the single-nucleus gene expression data of 41 post-mortem tissue samples from the prefrontal cortex and anterior cingulate cortex of 19 ASD patients and 16 control individuals [9]. We applied sparse module activity factorization (SMAF) [12] to gain modules with high independence and low redundancy based on random samplings of gene expression data of ASD and controls. Through statistical methods, modules that were significantly associated with ASD and highly reproducible were retained. Then, we identified cell-type-specific ASD-associated gene modules via the enrichment analysis between modules and cell markers. We verified these modules by the enrichment analysis with SFARI genes. Finally, we built ASD predictive models based on these gene modules and tested their performance. Our study provides new ideas for the localization of pathogenic factors of ASD and a new way to explore the mechanism of action of related genes in the pathogenesis, diagnosis, and clinical treatments of ASD.

## 2. Materials and Methods

### 2.1. Ethical Statement

This study was exempt from ethical approval as the used dataset is publicly available.

### 2.2. Single-Nucleus RNA-seq Data of ASD

We downloaded the raw counts of single-nucleus RNA-seq data of 19 ASD patients and 16 control individuals [9] from the website of autism.cells.ucsc.edu, which includes 104,559 nuclei from 41 post-mortem tissue samples from the prefrontal cortex and anterior cingulate cortex. The data contain 17 cell types, including fibrous astrocytes (AST-FB), protoplasmic astrocytes (AST-PP), endothelial, parvalbumin interneurons (IN-PV), somatostatin interneurons (IN-SST), SV2C interneurons (IN-SV2C), VIP interneurons (IN-VIP), layer 2/3 excitatory neurons (L2/3), layer four excitatory neurons (L4), layer 5/6 corticofugal projection neurons (L5/6), layer 5/6 cortico-cortical projection neurons (L5/6-CC), microglia, maturing neurons (Neu-mat), NRGN-expressing neurons I (Neu-NRGN-I), NRGN-expressing neurons II (Neu-NRGN-II), oligodendrocytes, and oligodendrocyte precursor cell (OPC). We preprocessed the data with R package “scran” [13], including the quality control of nuclei and genes, removing a minority of nuclei from different cell cycle phases, and normalizing the gene expression data. We used ComBat to regress out the covariates and technical factors that may contribute to the heterogeneity of gene expression, including age, sex, PMI (post-mortem interval), RIN (RNA integrity number), and sequencing batch. Nuclear and mitochondrial genes downloaded from Human MitoCarta2.0 were excluded [14]. Then, we obtained the final expression data involving 34,686 genes and 85,267 nuclei.

### 2.3. Data Set Partitioning and Matrix Factorization

For each cell type, the expression matrix $X\in R^{g\times S}$ was randomly divided into a training set and a testing set by the ratio of 7:3 for *N* times (*N* was set to ten in this study). We denoted the training set as $X_{trn}\in R^{g\times s}$, and denoted the corresponding testing set as $X_{ts}\in R^{g\times \left(S-s\right)}$ in a single division. We conducted the sparse module activity factorization (SMAF) [12] to calculate gene modules for each training set. The primary process is shown as:

(1) $$X_{\mathrm{trn}}\approx UW$$

The $U\in R^{g\times m}$ matrix is a non-negative, sparse “module dictionary”. Each column of the matrix represents a gene module, and each row of it represents the expression level of a gene in all modules. The $W\in R^{m\times s}$ is a matrix representing the activation levels of gene modules in cells. Each column of the matrix *W* represents a cell, and each row represents the activation level of a particular module in all cells. For our research, the modules we wished to find are related to ASD and have functional uniqueness. Since the original intention of SMAF is to serve for the recovery of high-dimensional data in compressed sensing, the matrix factorization result of SMAF has the following characteristics: (1) for the module dictionary matrix, to reduce the redundancy between modules, SMAF uses L1-relaxation to ensure the module’s sparsity; (2) for the module activation coefficient matrix, the SMAF algorithm forcibly restricts a maximum number of modules that would be activated for each cell, denoted by *K*. *K* was set to 15 in our research. These two features guarantee the uniqueness and low redundancy of the gene expression patterns among the modules resulting from SMAF. The sparse modules are also in line with the characteristics of single-nucleus gene expression data. Other parameters needed in the SMAF algorithm were set as default values in our research.

### 2.4. The Significance Tests of the Correlation Between Gene Modules and ASD

For all gene modules calculated by SMAF, we used two statistical methods to screen out gene modules associated with ASD. The first method was to screen the gene modules with significant differences in the distribution of module activation coefficients between ASD samples and control samples. To this end, we extracted the activation coefficients of a module from the matrix $W\in R^{m\times s}$, which is an individual row of *W* denoting by $W_{i,*},\;i\in \left\{1,2,\ldots ,m\right\}$ for the module *i*. It was supposed that among the *s* cells in $W\in R^{m\times s}$, the cells 1, 2, …, *s*_1_ were from the ASD group, and the cells *s*_1+1_, *s*_1+2_, …, *s* were from the control group. We denoted the module activation coefficients of ASD cells as $W_{a}=[W_{i,1},\;\ldots ,\;W_{i,s_{1}}]$, and denoted those in the control group as $W_{c}=\left[W_{i,s_{1+1}},\ldots ,\;W_{i,s}\right]$. Then, a T-test was performed between *W_a_* and *W_c_*. We calculated adjusted *p*-values by controlling the false discovery rate of multiple tests. For those modules with an adjusted *p*-value of T-test less than 0.1, it can be reckoned that there were significant differences in the distribution of activation coefficients between the ASD and control groups. Based on the results of the first method, the second statistical method selects the gene modules with a significant correlation between the module activation coefficient and the ASD/control label. We denoted the samples’ labels as vector *Y* = [*y*_1_, …, *y_s_*], *y_k_* ∈{+1, −1}, *k* ∈{1, 2, …, *s*}, where +1 indicates ASD samples and −1 indicates control samples. For a specific module *i*, *i* ∈{1, 2, …, *m*}, we calculated the Spearman correlations between *Y* and the module’s activation coefficients $W_{i,*}$. For those modules whose absolute value of Spearman correlation coefficients were larger than 0.1 and adjusted *p*-value less than 0.1, we deemed them as modules with a significant correlation between activation coefficient and samples’ label. The gene modules which passed the two kinds of tests are considered ASD-related gene modules.

### 2.5. Calculation of the Recurrent Rate of ASD-Related Gene Modules

We continued to pick out those modules with a highly recurrent probability to enhance the robustness of the results. Considering the heterogeneity of cell types, we would only calculate a particular module’s recurrent rate among gene modules obtained from the same cell type’s training sets. First, we determined the module genes by reserving the significantly expressed genes with z-score > 3. The reason for choosing this threshold is to keep the number of genes retained in all gene modules less than 1000. The modules’ significantly expressed genes were defined as module genes. For each module, the intersection of its module genes with another module was computed. Then, we calculated the percentage of this intersection to the number of module genes to obtain a recurrent rate. As mentioned before, for each cell type, there were *N* data partitions. For a data partition *n* ∈{1, 2, …, *N*}, we supposed there were *Kn* modules (*M_n_*_,1_, …, *M_n_*_,*Kn*_) and denoted *m_n_*_,*i*_, *i* ∈{1, 2, …, *Kn*} as the number of module genes in a specific module *M_n_*_,*i*_. Then, we defined *Mrr_n_*_,*i,o*_, *n,o* ∈{1, 2, …, *N*}, *n*≠*o*, as the maximum recurrent rate of module *M_n_*_,*i*_ in a partition *o*. *Mrr_n_*_,*i,o*_ was computed by:

(2) $${\mathrm{Mrr}}_{n,i,o}=\max\left[\frac{\mathrm{intersect}\left(M_{n,i},{\;M}_{o,1}\right)}{m_{n,i}},\;\ldots ,\;\frac{\mathrm{intersect}\left(M_{n,i},{\;M}_{o,\mathrm{Ko}}\right)}{m_{n,i}}\;\right]$$

where *n*, *o* ∈{1, 2, …, *N*}, *n*≠*o*, *i* ∈{1, 2, …, *Kn*}, $intersect\left(M_{n,i},M_{o,j}\right)$, *j* ∈{1, 2, …, *Ko*} calculates the number of shared genes between *M_n,i_* and *M_o_*_,*j*_. For any partition *o* except *n*, i.e., *o* ∈{1, 2, …, *N*}, *n* ≠ 0, if *Mrr_n_*_,*i,o*_ was greater than a given threshold, module *M_n_*_,*i*_ was considered highly reproducible.

### 2.6. Gene Enrichment Analysis

To further screen the gene modules specific to particular cell types, hypergeometric enrichment tests were applied between module genes (z-score > 3) and cell markers. For obtaining cell markers of each cell type, we applied the “findmarkers” function from the R package “scran” [13] with default parameters on the original gene expression matrix before data partitioning. For each cell type, the output was a sorted gene list, from which those genes that rank in the front could represent specific genes expressed in this cell type. We took the top 100 genes in the gene list as cell markers. If a module was significantly enriched with its cell markers, we deemed it as a cell-type-specific module. After identifying ASD-associated gene modules with cell-type specificity, we assessed their enrichment of known ASD candidate genes from the Simons Foundation Autism Research Initiative (SFARI) database [4]. To accurately assess the concealed genetic mechanism, SFARI assigns a score to each gene in the database to reflect the strength of evidence of the gene linking to the development of ASD. The human gene module in the SFARI database provides researchers worldwide with instant access to all known human genes related to ASD.

### 2.7. Construction of ASD Predictive Model

We applied the extreme gradient boosting (XGBoost) algorithm [15] to build the ASD diagnostic model using R package “xgboost” [15], with features derived from module genes in the highly reproducible ASD-related cell-type-specific modules. Compared with traditional gradient boosting decision tree (GBDT) [16], the XGBoost model explicitly includes a regularization term to control the model’s complexity, preventing overfitting, thus improving its generalization ability. When setting xGBoost model parameters, we set most parameters as their default values. For instance, we set the decision tree’s maximum depth to the default value of six, and the ratio of training samples was set to 0.5, meaning that XGBoost randomly collected half of the data instances to generate the decision tree, preventing overfitting. However, instead of using the default value of three for learning rate *eta*, we chose a more rigorous learning rate by setting it to 0.01, which controlled each decision tree’s contribution rate to the model. A lower learning rate could make the process more conservative, boosting the model’s robustness and preventing overfitting. At the same time, we set the number of iterations as ten.

## 3. Results

### 3.1. Overall Analytical Workflow

**Figure 1:**
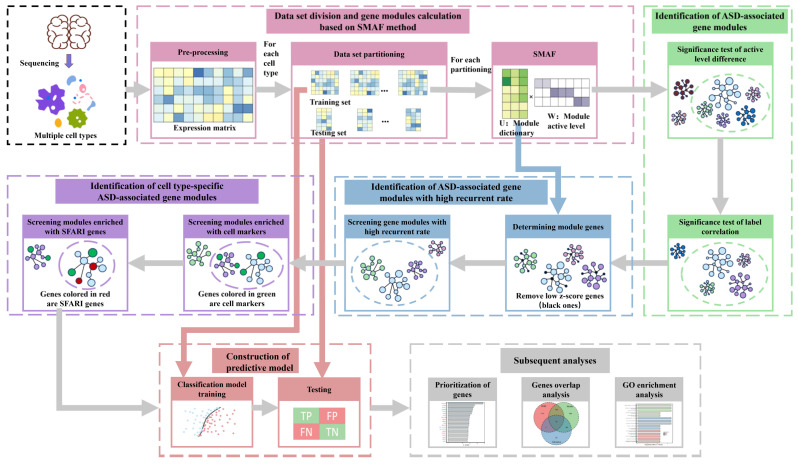
The overall workflow of screening cell-type-specific autism spectrum disorder (ASD)-associated gene modules.

In this study, an analytical workflow was established to analyze the brain single-nucleus gene expression data of 19 ASD patients and 16 control individuals [9] for acquiring ASD-associated gene modules with cell-type specificity via matrix factorization and statistical screening (Figure 1). The single-nucleus gene expression data was derived from 41 post-mortem tissue samples from the prefrontal cortex and anterior cingulate cortex, including 17 cell types identified by unbiased single-nucleus RNA sequencing [9]. The information about the individuals, including age, sex, post-mortem interval (PMI), and Autism Diagnostic Interview–Revised (ADI-R) scores that measured the impairments of behavioral domains in ASD (categories A, B-verbal, B-nonverbal, C and D) can be seen in Supplementary Table S1. Via data preprocessing, we obtained the gene-cell expression matrix (Materials and Methods). We divided each cell type’s expression matrix into training sets and testing sets by random sampling and repeated the procedure several times (Materials and Methods). We used each training set to perform SMAF matrix factorization (Equation (1)) to get a gene module matrix (*U*) and a module activation coefficient matrix (*W*). The following two statistical screenings were conducted on the matrix *W* to identify ASD-associated gene modules: (1) the significance test of the difference in the distribution of module activation coefficients between the ASD/control groups; and (2) the significance test of the correlation between module activation coefficients and ASD/control labels. Then, we statistically picked out gene modules with high recurrent rates (Equation (2)) within the data partitions of the same cell type to eliminate the randomness and contingency of data partitioning and matrix factorization. To further identify the cell-type-specific ASD gene modules, hypergeometric enrichment tests were carried out on these gene modules’ significantly expressed genes with cell markers and SFARI genes. Subsequently, we constructed ASD/control classification models using these gene modules based on the training sets and then executed ASD prediction on the test sets of the corresponding data partition. At the end, we performed GO (Gene Ontology) analysis on these cell-type-specific ASD-associated gene modules and prioritized genes to reveal their biological functions associated with ASD.

### 3.2. Identification of ASD-Associated Gene Modules

After data preprocessing and multiple partitioning for each cell type’s expression matrix, we obtained training sets $X_{trn_{i}}\in R^{g\times s},\;i\in \left\{1,2,\ldots N\right\}$. Here, *N* was set to ten. Then, based on each training set, SMAF was applied to decompose the matrix $X_{trn_{i}}$ into the product of a gene module matrix $U\in R^{g\times m}$ and a module activation coefficient matrix $W\in R^{m\times s}$. For the gene module matrix *U*, *g* is the number of genes, and *m* is the number of modules. Each column of *U* represents a gene module, and each row of *U* is a positive number vector representing the expression values for a particular gene in every module. Previous studies [17,18,19] have proven that modules generated by SMAF are of high independence and low redundancy. For the module activation coefficient matrix *W*, *m* is the module number, and *s* is the number of cells in this particular training set. Each row of *W* represents the activity level for a given module in every cell. We can see modules being active or not for an individual cell by checking a column of matrix *W*. The maximum number of activated modules for a cell can be preset in the SMAF algorithm, which was set to 15 in this study. We used SMAF to decompose each training set into 500 modules by setting *m* to 500.

**Figure 2:**
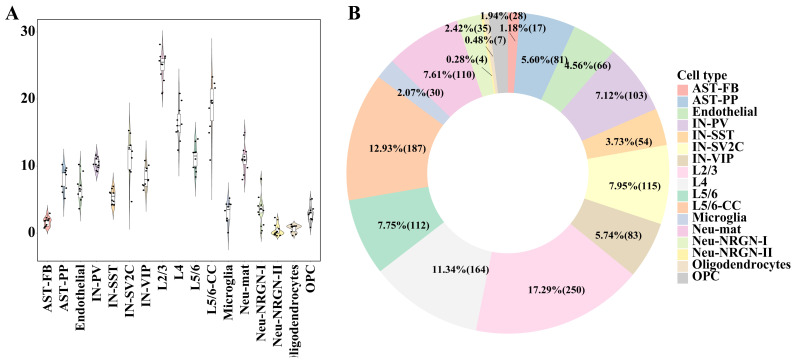
Distribution of the number of ASD-associated gene modules in each cell type. (**A**) Violin diagram of the number of ASD-associated gene modules obtained in each data partition for each cell type. Every dot denotes the gene modules’ number in each data partition. (**B**) The pie chart of the number of ASD-related gene modules. The pie chart percentage indicates the ratio of the gene modules’ number of each cell type to the total number of modules, followed by the modules’ number in brackets.

To identify the gene modules associated with ASD in the module dictionary *U*, we used two different statistical methods to test the *W* matrix’s corresponding rows: $W_{i},\;i\in \left\{1,2,\ldots m\right\}$. The first one was the significance test of the difference in module activation coefficients between ASD/control groups. The second was the significance test of the Spearman correlation between module activation coefficients and ASD/control labels (Materials and Methods). The gene modules that met the following conditions were considered ASD-associated gene modules: (1) adjusted *p*-values were less than 0.1 in both tests; (2) the absolute values of Spearman correlation coefficients were greater than 0.1 in the second test. It can be seen that for different cell types, the numbers of ASD-associated gene modules were dramatically different (Figure 2A,B). Generally speaking, the cell types with the most ASD-associated modules were excitatory neurons (L2/3, L5/6-CC, and L4). The number of ASD-associated gene modules obtained from the L2/3 cell type in each data partition was prominently greater than those obtained from other cell types, and the cell types that had the second and third largest number of modules were L5/6-CC and L4, indicating that excitatory neurons are the cell types most affected by ASD.

### 3.3. Identification of ASD-Associated Gene Modules with High Recurrent Rates

**Figure 3:**
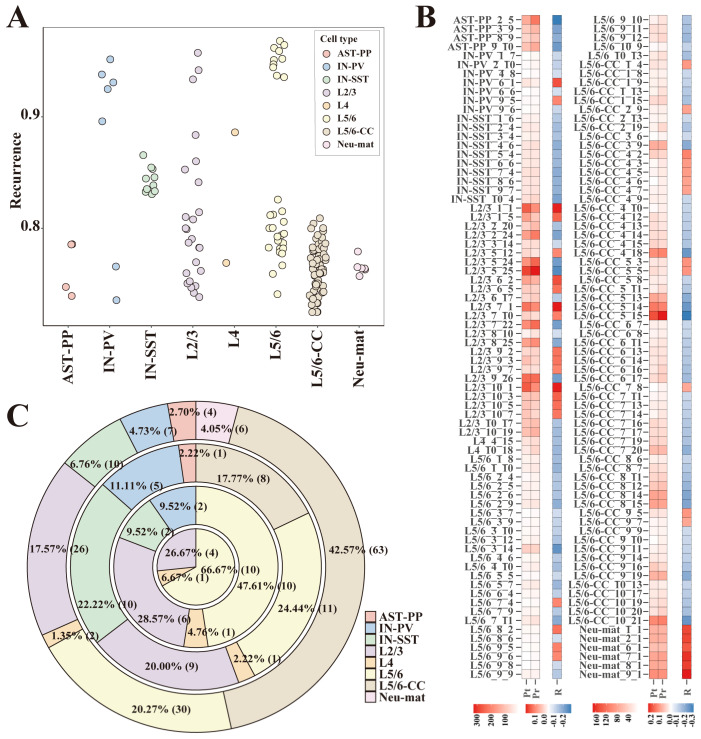
Gene modules with high recurrent rates. (**A**) Scatter plots of reproducible rates of the 148 gene modules with a reproducible rate greater than 70%. (**B**) The heat map of ASD-correlation tests for the 148 gene modules: the columns “Pt” and “Pr” show the −log 10 (adjusted *p*-value) of the two significance tests: (1) the significance test of the difference in module activation coefficients between ASD/control groups; and (2) correlations between the module’s activation coefficient and the cells’ ASD/control label. The “R” column displays Spearman correlation coefficients for the second test mentioned above. (**C**) The ring-shaped percentage graph of the number of gene modules when the recurrence threshold was 70%, 75%, 80%, and 85% (from the outside to the inside). Note: gene module indexes are composed of: the module’s cell type, the sequence number of the data partition, and the sequence number of the module in the data partition.

After ASD-related gene modules were recognized, to eliminate random factors in the matrix factorization process, all ASD-associated gene modules’ recurrent rates were calculated (Materials and Methods). We hypothesized that the module genes were not gathered together accidentally for those gene modules with high recurrent rates. To screen out highly reproducible modules, first, we calculated the z-score values of all genes in each module and deemed those genes with high z-score values as significantly expressed genes. The module genes were determined as significantly expressed genes. Then, by adjusting the threshold of z-score values, the number of significantly expressed genes of all modules could be less than 1000. For each module, if the maximum recurrent rate of a module in all data partitions from its cell type was greater than the preset threshold, we deemed it a reproducible module (Materials and Methods). When the recurrent rate threshold was set to 70%, 148 highly reproducible gene modules from eight different cell types were screened (Figure 3A). These 148 modules passed the two kinds of significance tests described in the previous section (Figure 3B) and had low contingency during data partition and matrix factorization. Since the recurrent rates were calculated within gene modules of the same cell type, to a certain extent, these modules are cell-type-specific. As we can see in the screening results, the excitatory neurons like L2/3, L5/6, and L5/6-CC dominated the number of reproducible gene modules (Figure 3A,C). When we gradually increased the threshold of recurrent rates (70%, 75%, 80%, and 85%), the proportion of gene modules from the excitatory neurons gradually increased. When the threshold of recurrent rates was increased to 85%, the remaining gene modules were only from excitatory neurons (L2/3, L4, L5/6) (Figure 3C).

### 3.4. Identification of Cell-Type-Specific ASD-Associated Gene Modules

**Figure 4:**
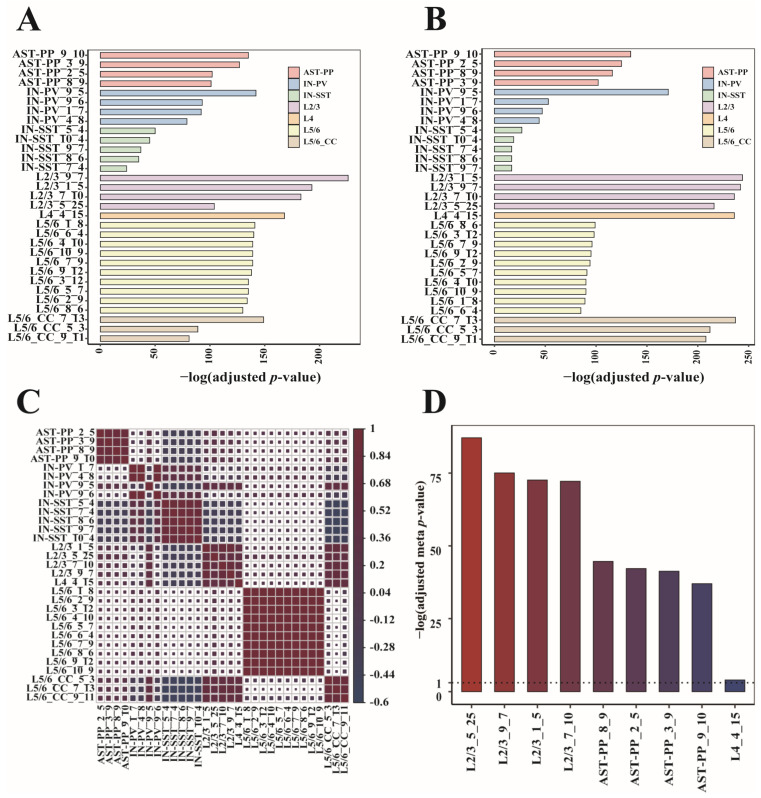
Enrichment and correlation analysis of the 31 cell-type-specific ASD-associated gene modules. The bar plot of −log 10 (adjusted *p*-values) of enrichment analysis between each gene module and (**A**) cell markers and (**B**) Simons Foundation Autism Research Initiative (SFARI) genes. (**C**) The Spearman correlation diagram of the 31 modules. (**D**) The −log 10 (adjusted meta *p*-values) of cell-type-specific ASD-associated gene modules that significantly correlated with clinical severity of ASD.

We conducted a further statistical calculation to identify gene modules with cell-type specificity. Firstly, we obtained the differentially expressed genes representing each cell type. For this purpose, we used the “findmarkers” function of the R package “scran” [13], whose input is the original gene expression matrices of all cell types, and output is a sorted gene list for each cell type. The top 100 genes in every sorted gene list were served as cell markers. Then, we performed hypergeometric enrichment tests on the module genes and cell markers (Materials and Methods). Modules that were mostly enriched with cell markers of their cell types were considered cell-type-specific. We got 31 gene modules covering seven cell types (Figure 4A). When looking at each gene module’s enrichment result, the gene modules most enriched with the cell markers were from L2/3, followed by L4 and L5/6-CC (Figure 4A). To verify these gene modules’ correlations to ASD, we implemented the hypergeometric enrichment test on these gene modules and SFARI genes. It can be seen that the 31 gene modules were all significantly enriched with the SFARI genes, and the gene modules mostly enriched with the SFARI genes were still from L2/3, followed by L5/6-CC and L4 (Figure 4B). To examine the correlations among the 31 modules, we extracted the corresponding columns of these modules in their *U* matrixes, assigned the expression values to zeroes for the non-significantly expressed genes, and then calculated the Spearman correlation coefficients of these columns. We can see that gene modules from the same cell type have strong correlations, proving that these gene modules are cell-type-specific (Figure 4C). Next, we checked whether these cell-type-specific ASD-associated gene modules were correlated with the clinical severity of ASD. We ranked the ADI-R scores of patients within each ADI-R category and used the sum of ranks as the final clinical severity score of each patient. For each gene in each cell-type-specific ASD-associated gene module, we calculated the patient-level fold change of gene expression by comparing each ASD patient to the control group. Then, we correlated patient-level fold changes with patient clinical severity scores to calculate Pearson’s correlation coefficient and associated *p*-value. Using the *p*-values of all module genes, we determined the meta *p*-value for each cell-type-specific ASD-associated gene module using Fisher’s method. Then, the meta *p*-values were adjusted by controlling the false discovery rate of multiple tests. We observed that changes in all ASD-associated gene modules specific to L2/3, AST-PP, and L4 were correlated with clinical severity of ASD (Figure 4D), indicating that these cell types are predictive of clinical severity.

### 3.5. Prediction of ASD Based on Cell-Type-Specific ASD-Associated Gene Modules

**Figure 5:**
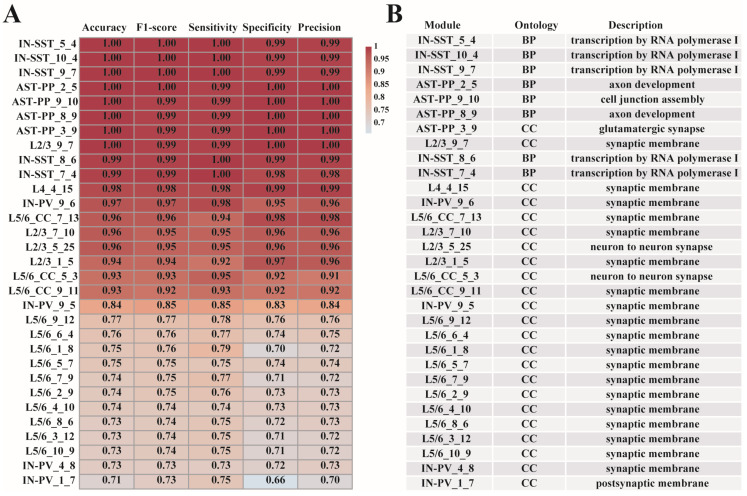
(**A**) The predictive performance and (**B**) the top one enriched GO functions of the final 31 cell-type-specific ASD-associated gene modules.

To check whether the 31 gene modules have predictive effects for ASD, we used the xgboost method [15] to construct cell-type-specific ASD predictive models based on the module genes. To prevent overfitting, we introduced a regularization term into the models (Materials and Methods). For each gene module, we used the data division’s training set from which the module was generated to build the model and the matching test set for model testing (Figure 1). The results show that the predictive model established by the modules we screened has excellent ASD/control classification capabilities. All models have a predictive accuracy more than 70%, and more than half of the models (19 out of 31 modules) have a predictive accuracy above 80% (Figure 5A). The 19 modules are from AST-PP, IN-SST, IN-PV, L2/3, L4, and L5/6-CC cell types. The excellent predictive performances of these models also confirmed the significant correlations between the gene modules and ASD. The genes contained in the 31 cell-type-specific ASD-associated gene modules can be seen in Supplementary Table S2. Next, we continued to analyze the specific relationships between these modules and ASD via GO function enrichment analysis using R package “clusterProfiler” [20]. The enriched GO functions of the 31 gene modules (adjusted *p*-value < 0.1) are listed in Supplementary Table S3. The respective top one enriched GO functions of the 31 modules are also shown in Figure 5B. We can explore the cell-type heterogeneity among these different modules and study how these functions were connected to ASD. For example, by checking the top one GO term enriched with each gene module, we found that functions such as cell junction assembly, axon development, and glutamatergic synapse were significantly enriched in modules from AST-PP cells, while transcription by RNA polymerase I were significantly enriched in modules from IN-SST cells.

### 3.6. Prioritization of Genes in Cell-Type-Specific ASD-Associated Gene Modules

**Figure 6:**
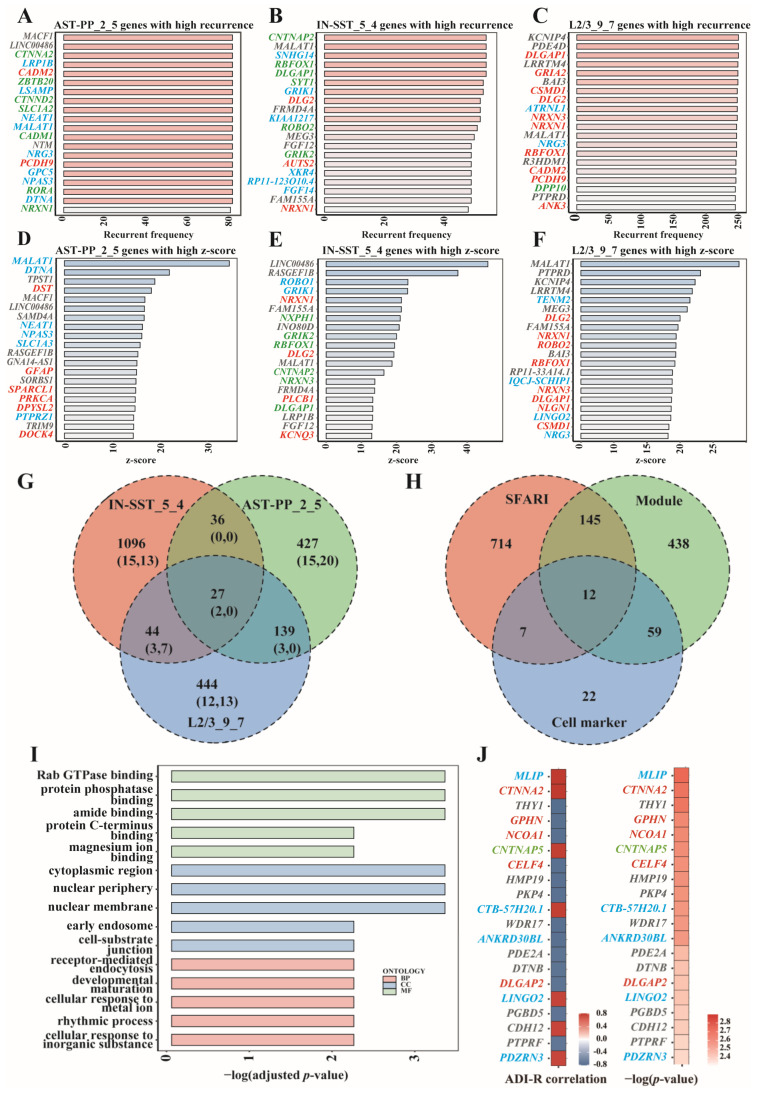
Comprehensive analysis of gene modules AST-PP_2_5, IN-SST_5_4, and L2/3_9_7. (**A**–**C**) The bar plot of the top 20 recurrent frequency genes in modules AST-PP_2_5, IN-SST_5_4, and L2/3_9_7. SFARI genes in red, cell markers in blue, and the intersection of the two in green. (**D**–**F**) The bar plot of the top 20 z-score genes in modules AST-PP_2_5, IN-SST_5_4, and L2/3_9_7. SFARI genes in red, cell markers in blue, and the intersection of the two in green. (**G**) Venn plot of genes in modules AST-PP_2_5, IN-SST_5_4, and L2/3_9_7. The number outside the bracket indicates module genes, the first number in the bracket indicates top 20 recurrent frequency genes, and the second number indicates top 20 z-score genes. (**H**) Venn plot of the module genes in module L2/3_9_7, cell markers, and SFARI genes. (**I**) Functional analysis of gene module L2/3_9_7. (**J**) The Pearson’s correlation coefficients and associated *p* values of the top 20 genes which are correlated with ASD clinical severity in module L2/3_9_7. SFARI genes in red, cell markers in blue, and the intersection of the two in green.

From the predictive performance of these gene modules, we can see that IN-SST_5_4, AST-PP_2_5, and L2/3-CC_9_7 were the top modules of interneurons, astrocytes, and excitatory neurons. To analyze the cell-type specificity of these modules, we compared the top genes of the above three modules. We listed the top genes according to the genes’ recurrent frequency and their z-score values and marked the cell markers and SFARI genes. It can be seen that among the top 20 genes ranked by recurrent frequency, AST-PP_2_5 had five common genes with L2/3_9_7 and two common genes with IN-SST_5_4; IN-SST_5_4 had five common genes with L2/3_9_7; there were two common genes among these three modules, namely *MALAT1* and *NRXN1* (Figure 6A–C,G). *NRXN1* is one of the SFARI genes [4], while *MALAT1* was proven to be a risk gene for various cancers such as non-small cell lung cancer, hepatocellular carcinoma, gastric cancer, and pancreatic cancer [21,22]. Among the top 20 z-score value genes, AST-PP_2_5 had no common gene with L2/3_9_7 and IN-SST_5_4; IN-SST_5_4 had seven common genes with L2/3_9_7 (Figure 6D–G). These results are consistent with the cell-type specificity requirement when we screened modules. We further looked at the module genes in these modules and also found that they differ significantly (Figure 6G).

Noting that L2/3 is the cell type that was most enriched with cell markers and SFARI genes, we then looked at L2/3_9_7 carefully. Both the top 20 genes of L2/3_9_7 ranked by z-score and recurrence rate had significant overlap with SFARI genes and cell markers (Figure 6C,F). Overall, 654 genes were significantly expressed in L2/3_9_7, of which 157 belonged to SFARI, and 71 could be used as cell marker genes (Figure 6H). The L2/3_9_7 module’s GO analysis results involved functions such as protein phosphatase binding, early endosome, and developmental maturation that may affect neuronal cells’ development and status in the brain. (Figure 6I). Among the overlapping genes between top 20 recurrent frequency genes and top 20 z-score genes in L2/3_9_7, *MALAT1*, *NRG3*, *KCNIP4*, *BAI3*, *PTPRD*, and *LRRTM4* were not included in the SFARI ASD genes. Except *MALAT1*, a risk gene of multiple kinds of tumors [21,22], there is evidence showing the associations between the other genes and ASD or other neurological diseases. Gene *NRG3* is a cell marker gene of L2/3, and it has also been shown to be associated with ASD. Previous research indicated that *NRG3* is an ASD candidate gene that showed allele-biased expression in the brains of ASD patients. Such allele-biased expression may lead to neuronal differentiation and neuropsychiatric disorders [23]. Another study revealed that the expression level of *NRG3* dramatically increased in activated microglia in ASD patients’ brains. The positive correlation between the expression level of *NRG3* and clinical manifestations of children with ASD suggested that *NRG3* was involved in ASD’s pathobiology [24]. Gene *KCNIP4* is related to calcium ion binding and potassium channel regulatory activity. Bioinformatics analysis showed that *KCNIP4* was involved in neurite growth, synaptic plasticity, neuron proliferation, and neuron differentiation, which were considered to be associated with attention-deficit/hyperactivity disorder (ADHD) [25]. Studies have also shown that *KCNIP4*, as a gene encoding potassium channels, plays a vital role in maintaining membrane potentials of different neurons in different potassium channels. Erroneous regulation of these neurons is associated with intellectual diseases such as fragile X syndrome (FXS) [26]. During neuron differentiation, potassium ion channels’ attenuation would lead to the impairment of neuron function caused by neuron immaturity [26]. *BAI3* regulates many aspects of the central nervous system, including axon guidance, myelin formation, and synapse formation. Variation of the *BAI3* gene may cause cognitive impairment and ataxia. *BAI3*-encoded proteins play essential roles in mice’s neurodevelopmental processes, influencing the clinical symptomatology of schizophrenia [27]. *BAI3* is also highly expressed in hippocampal neurons and plays a vital role in regulating synaptic density. A reduction of *BAI3* in hippocampal neurons severely impairs dendritic morphogenesis in mice, leading to symptoms such as emotional instability, anxiety, and social closure [28]. *PTPRD* is a receptor protein tyrosine phosphatase, which is genetically related to neurodevelopmental disorders. The loss of *PTPRD* will increase intermediate progenitor cells and cortical neurons and the disturbance of neuronal localization. The loss of *PTPRD* will also lead to the overactivation of neural precursors and their downstream signaling pathways. These results suggest that *PTPRD* regulates receptor tyrosine kinases to ensure an appropriate number of intermediate progenitor cells and neurons associated with neurodevelopmental disorders’ genetic mechanisms [29]. *PTPRD* is also related to synaptic differentiation in the brain. Deleting one of the *PTPRD* alleles in mice leads to memory impairment and altered electrophysiological responses in the hippocampus. Moreover, *PTPRD* is considered one of the candidate genes for Alzheimer’s disease. In a Drosophila model, the knockout of *LAR* (a drosophila homolog of *PTPRD*) can lead to age-related retinal degeneration [30]. As to *LRRTM4*, a protein coding gene, its related pathways include protein-protein interactions at the synapses and neuronal chemical transmembrane transmission. Original *LRRTM4* contains multiple hormonal response parts (HREs). Variations within these HREs may alter the binding and activation of different hormone receptors, leading to an increased risk of mental disease [31]. *LRRTM4* is essential for brain synaptic production activity. The decreasing expression of *LRRTM4* protein in the hippocampus caused by aging leads to 15-month-old rats’ habituation of acoustic startle response (ASR) and impairment of learning and memory in the maze experiment [32]. The *LRRTM-NRX-Hs-PTPS* synaptic complex is a molecular center, and its destruction may be one of the causes of ASD [33].

L2/3_9_7 is also correlated with clinical severity of ASD (Figure 4D). For the top 20 genes which are correlated with ASD clinical severity, we demonstrated their Pearson’s correlation coefficients and associated *p*-values (*p* < 0.005) (Figure 6J). Among these top 20 genes, six belonged to SFARI genes, including *CTNNA2* (category S), *GPHN* (category 2), *NCOA1* (category 1), *CNTNAP5* (category 3), *CELF4* (category 1), and *DLGAP2* (category 3). Moreover, gene *LINGO2* was noted, which is also in the list of top 20 z-score genes. *LINGO2* encodes a transmembrane protein mainly expressed in the central nervous system. The single nucleotide polymorphisms in intronic regions of *LINGO2* has been verified to be linked to essential tremor and Parkinson’s disease, which are neurodegenerative disorders [34].

## 4. Discussion

Considering the multifactorial and cell-type-specific characteristics of ASD pathogeneses, we performed multiple random partitioning on the single-nucleus expression matrix of ASD patients and healthy individuals for each cell type. We then used the sparse decomposition method SMAF to obtain gene modules from the training set in each data partition. By two statistical procedures, we screened out ASD-related modules for each cell type. The number of ASD-related modules obtained from each cell type in a single data partition varies greatly. The top three cell types with the most ASD-related gene modules are L2/3, L5/6-CC, and L4. To obtain robust ASD-associated gene modules, we calculated their recurrent rates. Then, 148 ASD-associated modules with recurrence rates greater than 70% were identified across eight cell types (AST-PP, IN-PV, IN-SST, L2/3, L4, L5/6, L5/6-CC, and Neu-mat). The top three cell types with the largest number of gene modules with high recurrent rates were L5/6-CC (63, 42.57%), L5/6 (30, 20.27%), and L2/3 (26, 17.57%), suggesting the excitatory neurons may be most related to ASD. By performing enrichment analysis of cell markers, 31 modules were identified as cell-type-specific ASD-associated gene modules, covering seven types of cells (AST-PP, IN-PV, IN-SST, L2/3, L4, L5/6, and L5/6-CC). Among the 31 modules, the modules most enriched with cell markers were from L2/3, followed by L4 and L5/6-CC, and the modules most enriched with SFARI genes were still from L2/3, followed by L5/6-CC and L4, indicating that the cells most closely related to ASD may come from excitatory neurons. Previous research has shown that excessive excitatory cortical neurons produced before birth can lead to extreme brain growth imbalance, abnormal stimulation and inhibition, abnormal formation of the cortex, and further abnormal social interaction and behavior [35]. Pieces of evidence suggest that ASD’s pathogenic processes involve an imbalance between excitability and inhibition in the cerebral cortex, associating with excess excitatory neurons [3].

Our results are highly consistent with Velmeshev’s research [9]. Velmeshev’s study showed that ASD-related genes were enriched in astrocytes and synaptic functions displayed common shear changes in interneurons, explaining why the 31 modules we identified involved AST-PP, IN-PV, and IN-SST cells. The correlation analysis of the 31 modules showed that the correlation of modules within a cell type is stronger than the correlation of modules between different cell types, confirming the cell-type specificity of ASD pathogenic factors emphasized in Velmeshev’s study [9,11]. Through GO analysis of these 31 gene modules, it was found that their significantly enriched GO functions involve functions like postsynaptic membrane, synaptic membrane, transcription by RNA polymerase I, and neuron to neuron synapse, which are related to the pathogenic factors of ASD.

We used the 31 gene modules to build cell-type-specific ASD predictive models that could excellently classify cells between ASD patients and healthy individuals. Among the genes significantly expressed in these modules, those with the highest recurrent frequency have a great chance to own the highest z-score values. In addition to SFARI genes, other genes with top 20 recurrent frequency and top 20 z-score values have mostly been linked to ASD or other neurological diseases in the latest studies. Take the L2/3_9_7 module as an example: *NRG3*, *KCNIP4*, *BAI3*, *PTPRD*, and *LRRTM4* belonging to protein-coding genes are prioritized. The functions of these five genes cover synapses, neuron proliferation, protein interaction, and chemical transmission in the synapses. Their mutations and dyscontrol have also been proven to be associated with various mental disorders, including schizophrenia, ataxia, depression, and ASD [26,28,29,30,31,32,33,35]. When checking the association between genes and clinical severity of ASD, *LINGO2* is of note, which has been verified to be linked to neurodegenerative disorders, such as essential tremor and Parkinson’s disease. These genes are likely to play critical roles in the pathogenesis of ASD and may become new therapeutic targets for ASD. It is of great significance to study the change of expression intensity, gene interactions, and differentiation of the identified gene modules in ASD patients’ cells, which is helpful to develop ASD treatment targets and understand the pathogenesis of ASD. Our approach based on gene module analysis is a general analytical framework for studying the cell-type-specific multi-gene sets associated with complex diseases.

## 5. Conclusions

Intending to explore the cell-type-specific pathogenic factors of ASD, we identified highly reproducible ASD-associated gene modules based on matrix decomposition and statistical tests. Then, we further detected the ones which are cell-type-specific and significantly related to ASD. The predictive models built based on these cell-type-specific ASD-associated modules have demonstrated excellent performance. Our analytical method offers new ideas for identifying potential pathogenic factors and exploring multiple genes’ interrelationships in complex diseases, providing new potential therapeutic target genes, clinical diagnosis, and treatment for ASD. Our approach can be applied to other complex diseases involving multiple gene interactions and cellular heterogeneity.

## Data Availability

The raw counts of human brain single-nucleus RNA-seq data of ASD and controls was downloaded from: autism.cells.ucsc.edu, and the processed data can be accessed at: https://doi.org/10.5281/zenodo.4641804.

## Supplementary Materials

The following are available online at https://www.mdpi.com/article/10.3390/biomedicines9040410/s1. Supplementary Table S1: The information of the ASD patients and control individuals, including age, sex, PMI, and ADI-R scores (categories A, B-verbal, B-nonverbal, C and D). Supplementary Table S2: The genes contained in the 31 cell-type-specific ASD-associated gene modules. The recurrent frequency and z-score values of genes in each module are also listed. Supplementary Table S3: The GO functions of the 31 cell-type-specific ASD-associated gene modules.
